# Development and validation of an MRI-Based nomogram to predict the effectiveness of immunotherapy for brain metastasis in patients with non-small cell lung cancer

**DOI:** 10.3389/fimmu.2024.1373330

**Published:** 2024-04-15

**Authors:** Junhao Xu, Peiliang Wang, Yikun Li, Xiaonan Shi, Tianwen Yin, Jinming Yu, Feifei Teng

**Affiliations:** ^1^ Department of Radiation Oncology, Shandong Cancer Hospital and Institute, Shandong First Medical University and Shandong Academy of Medical Sciences, Jinan, China; ^2^ Department of Radiation Oncology, Shandong Cancer Hospital and Institute, Cheeloo College of Medicine, Shandong University, Jinan, China; ^3^ Cancer Center, Union Hospital, Tongji Medical College, Huazhong University of Science and Technology, Wuhan, China

**Keywords:** immunotherapy, brain metastasis, non-small cell lung cancer, radiomics, MRI

## Abstract

**Introduction:**

The variability and unpredictability of immune checkpoint inhibitors (ICIs) in treating brain metastases (BMs) in patients with advanced non-small cell lung cancer (NSCLC) is the main concern. We assessed the utility of novel imaging biomarkers (radiomics) for discerning patients with NSCLC and BMs who would derive advantages from ICIs treatment.

**Methods:**

Data clinical outcomes and pretreatment magnetic resonance images (MRI) were collected on patients with NSCLC with BMs treated with ICIs between June 2019 and June 2022 and divided into training and test sets. Metastatic brain lesions were contoured using ITK-SNAP software, and 3748 radiomic features capturing both intra- and peritumoral texture patterns were extracted. A clinical radiomic nomogram (CRN) was built to evaluate intracranial progression-free survival, progression-free survival, and overall survival. The prognostic value of the CRN was assessed by Kaplan–Meier survival analysis and log-rank tests.

**Results:**

In the study, a total of 174 patients were included, and 122 and 52 were allocated to the training and validation sets correspondingly. The intratumoral radiomic signature, peritumoral radiomic signature, clinical signature, and CRN predicted intracranial objective response rate. Kaplan–Meier analyses showed a significantly longer intracranial progression-free survival in the low-CRN group than in the high-CRN group (*p* < 0.001). The CRN was also significantly associated with progression-free survival (*p* < 0.001) but not overall survival.

**Discussion:**

Radiomics biomarkers from pretreatment MRI images were predictive of intracranial response. Pretreatment radiomics may allow the early prediction of benefits.

## 1 Introduction

Non-small cell lung cancer (NSCLC) is the predominant form of lung cancer, accounting for around 80-85% of all reported cases (1). Immune checkpoint inhibitors (ICIs) targeting programmed cell death-1 (PD-1), programmed cell death ligand-1 (PD-L1), and cytotoxic T-lymphocyte antigen-4 (CTLA-4) have shown promising results in advanced NSCLC and prolonged patient survival. ICIs have transformed the therapeutic prospect of metastatic NSCLC (2, 3).

Approximately 25% of patients with NSCLC are diagnosed with brain metastases (BMs) (4), and 20–40% of patients develop BMs throughout their whole life (5, 6). Patients with NSCLC and BMs have a high mortality rate (7). Although ICIs have resulted in improvements in advanced NSCLC treatment, their effectiveness in treating metastatic brain lesions remains controversial (8). Patients with baseline BMs benefited from pembrolizumab treatment in the KEYNOTE-189 study, whereas other studies, such as KEYNOTE-024, found no benefit (9, 10). Among the patients who had undergone therapy for brain metastases in the KEYNOTE-024 trial, the pembrolizumab group exhibited a longer median progression-free survival (PFS) in comparison to the chemotherapy group; however, no statistically significant difference in survival outcomes between the immunotherapy and chemotherapy groups. In a phase II trial involving 37 patients diagnosed with asymptomatic BMs in NSCLC, only a minority (29.7%) of individuals with PD-L1-positive NSCLC exhibited a favorable response in terms of BMs (11). Previous clinical trials have demonstrated that immunotherapy could potentially be beneficial for patients with NSCLC with BMs, and biomarkers to select patients who could benefit from ICIs are required. Although the potential biomarkers for immunotherapy, including PD-L1 expression levels, tumor-infiltrating lymphocytes (TILs), and tumor mutation burden (TMB), have been investigated, none of them have shown a significant association with the intracranial response or prognosis (11–13).

New opportunities have arisen with the recent advent of radiomics and quantitative imaging biomarkers. Different from conventional biopsy-based tests that only capture a portion of the tumor, imaging provides a complete perspective of the entire tumor burden, offering valuable insights into each cancerous lesion through a single non-invasive examination (14). Radiomics-based biomarkers have been successful in predicting patient survival, tumor microenvironment status, and the differentiation of malignant tumors (15–18). However, little research has investigated the correlation between radiomics and intracranial advancement in patients diagnosed with NSCLC and BMs undergoing immunotherapy. In this retrospective study, we employed contrast-enhanced magnetic resonance imaging (CE-MRI) to develop a radiomics nomogram that can predict the effectiveness of intracranial immunotherapy in patients with NSCLC with BMs.

## 2 Materials and methods

### 2.1 Patients

**Figure 1:**
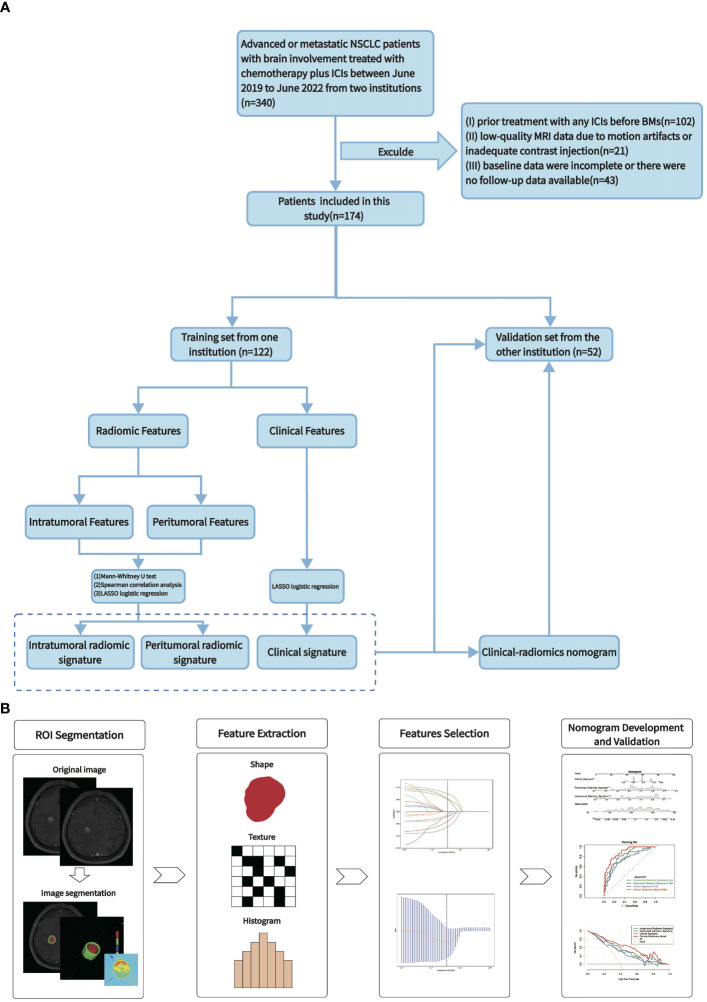
Study overview. **(A)** Workflow of the study. **(B)** Workflow of the radiomics analysis.

The study received approval from the medical ethics committee of the institutions, and the requirement for obtaining informed consent was exempted. We identified 340 advanced NSCLC patients with brain involvement who had received ICIs, including sintilimab, camrelizumab, tislelizumab, and pembrolizumab, between June 2019 and June 2022 at Shandong Cancer Hospital and Institution, and Cheeloo Hospital in Jinan, China. The inclusion criteria were (1) age ≥18 years; (2) Karnofsky Performance Status (KPS) ≥ 70; (3) baseline CE-MRI performed within 4 weeks before immunotherapy; and (4) BMs meeting the criteria for measurable disease according to Response Assessment in Neuro-Oncology Brain Metastases (RANO-BM) (≥ 0.5 cm) (19). Patients were excluded if they had: (1) prior treatment with any ICIs before being diagnosed with BMs; (2) low-quality MRI data due to motion artifacts or inadequate contrast injection; or (3) incomplete baseline data, or if there were no follow-up data available. The selected patients were separated into a training cohort from Shandong Cancer Hospital and Institution and an independent external validation cohort from Cheeloo Hospital. The recruitment process and exclusion criteria are shown in **Figure 1A**.

Baseline clinical characteristics, such as KPS score, age, gender, history of smoking, clinical stages, number and diameter of BMs, and blood indicators, were obtained from medical records, and imaging of baseline MRI were also downloaded. Two experienced neuroradiologists independently evaluated the quantifiable intracranial target lesions according to the RANO-BM guidelines and classified the intracranial response as good or poor, based on intracranial objective response rate (ORR) after 4 cycles of ICIs. If there was any conflict over the results of the assessment, another radiologist was invited to reach a final decision. Intracranial target lesions with any of the following were defined as having a good response: (1) intracranial complete response (iCR) or (2) intracranial partial response (iPR). The poor response was defined with any of the following: (1) intracranial progressive disease (iPD) or (2) stable disease (iSD). Continuous patient follow-up was performed throughout the study to ensure accurate ascertainment of survival outcomes. Intracranial progression-free survival (iPFS) pertains to the duration starting from the initiation of ICI treatment until the documented occurrence of brain progression or mortality due to any cause. Progression-free survival (PFS) is defined as the timeframe commencing from the first administration of ICI until the recorded manifestation of progression in any lesion or death resulting from any cause. Overall survival (OS) was determined by computing the period beginning with the initial application of ICI and ending at either demise or the most recent follow-up.

### 2.2 Magnetic resonance imaging acquisition

Patients from Shandong Cancer Hospital were scanned using an eight-channel phased-array surface coil on a 3.0T scanner system from Siemens (Magnetom Verio, Siemens, Erlangen, Germany) according to the protocols: Repetition Time (TR)=270ms; Echo Time (TE)=2.48ms; Slice thickness=5mm, Field of View (FOV) = 194×230mm, Matrix Size = 320×216. Patients from Cheeloo Hospital were scanned using an eight-channel phased-array surface coil on a 3.0T scanner system from GE (GE Signa HDX, USA), according to the protocols: TR = 1400ms; TE = 9ms; Slice thickness = 6mm, FOV = 179×230mm, Matrix Size = 320×187. Prior to the MRI, gadodiamide (0.1 mmol/kg, Omniscan, GE Healthcare) was intravenously injected using a power injector with a speed of 2.5 mL/s. Subsequently, 20 mL saline solution was injected at the same rate to clear any remaining contrast agent. The scanning range in the supine position encompassed the scalp and lower neck.

The radiomics analysis workflow comprised image segmentation, feature extraction, feature selection, model development, and model validation (**Figure 1B**).

### 2.3 Image segmentation and radiomic feature extraction

Two experienced neuroradiologists, unaware of the patient’s medical details, independently delineated the tumor region of interest (ROI) using ITK-SNAP (version 3.8.0, http://www.itksnap.org) on T1 contrast-enhanced MRI sequences (T1CE). The tumor area was delineated on the slice with the maximum tumor area, excluding peritumoral edema. The peritumor region was defined as the 5 mm area surrounding the tumor. Two ROIs were finally delineated for each metastatic brain lesion: (1) the intratumoral ROI, the whole metastatic brain lesion; and (2) the peritumoral ROI, extending 5 mm around the intratumoral region.

The radiomic features were obtained from the two ROIs utilizing Pyradiomics, an open-source Python package. Before feature extraction, MRI images are preprocessed, including image normalization, resampling, discretization, and filtering. Eight filters were used including Gaussian Laplacian, logarithm, wavelet, exponent, square, square root, ladder, and local binary mode.

### 2.4 Radiomic features selection and signatures constructed

The feature-selection process mentioned above was executed exclusively on a training set. After standardizing all radiomic features using z-score normalization, three steps were used to identify the optimal features of each ROI for predicting the intracranial response to immunotherapy. First, the Mann–Whitney U-test was performed to determine significant factors that distinguish patients in the good and poor response groups. Only the statistically significant features were retained for further analysis. Second, to eliminate redundant radiomic features, Spearman correlation analysis was performed, and features with a strong correlation coefficient (Spearman correlation coefficient > 0.9) were excluded. Finally, least absolute shrinkage and selection operator (LASSO) logistic regression with 5-fold cross-validation using the minimum criteria were performed. For predictive purposes, the intratumoral and peritumoral radiomic signatures were constructed using support vector machine (SVM) methods, by weighting the chosen characteristics from the two ROIs with their corresponding LASSO coefficients. Finally, two predictive models were constructed: the Intratumoral Radiomic Signature (IRS) and the Peritumoral Radiomic Signature (PRS).

### 2.5 Nomogram development and validation

The intracranial immunotherapy response was analyzed using LASSO logistic regression with 5-fold cross-validation and minimum criteria to identify clinically significant characteristics. Significant characteristics were weighted based on their respective LASSO coefficients and used SVM methods to construct Clinical Signature (CS). Subsequently, to offer a visually quantifiable tool for predictive purposes, a personalized clinical radiomics nomogram (CRN) was developed using a multivariable logistic regression algorithm in the training set, which effectively integrated intratumoral and peritumoral radiomics and clinical signatures. The prediction models were evaluated by analyzing the receiver-operating characteristic (ROC) curve and comparing them using the DeLong test on both the training and validation sets. The model performance was evaluated by calculating the average area under the curve (AUC) along with a 95% confidence interval (CI), sensitivity (SEN), specificity (SPE), accuracy (ACC), positive predictive value (PPV), and negative predictive value (NPV). To evaluate and compare the clinical efficacy of each model, decision-curve analysis (DCA) was employed. Additionally, the nomogram was assessed using the Hosmer-Lemeshow test, which quantified the correspondence between the predicted and observed probabilities in the CRN, followed by plotting a calibration curve. In order to evaluate the predictive prognostic significance of CRN, we calculated individual CRN scores for every participant and then classified them into high-risk (high CRN) and low-risk (low CRN) categories using the median CRN score as the threshold.

### 2.6 Statistical analysis

Chi-squared tests were utilized to assess the clinical characteristics between the training and validation cohorts in terms of categorical variables, while independent t-tests were employed for continuous variables. The statistical analyses were performed following established academic practices, utilizing R (version 4.2.0) and Python software (version 3.6.5). Statistically significant was defined as having two-sided *p*-values less than 0.05. To evaluate the differences in iPFS, PFS, and OS between the high-CRN and low-CRN cohorts, we performed Kaplan-Meier survival analysis along with log-rank tests.

## 3 Results

### 3.1 Patient clinical characteristics

After the implementation of the criteria for inclusion and exclusion, 174 cases were selected and stratified into two cohorts: (1) a training cohort (n = 122) from Shandong Cancer Hospital and Institution was used to construct the optimal prediction models, and (2) a validation cohort (n = 52) from Cheeloo Hospital was utilized to evaluate model performance and goodness-of-fit.

Of all the patients enrolled (n = 174), the median age was 59.0 years (range, 32−77 years) and 72.4% were male (126/174). Approximately 40% (70/174) of the patients had a KPS score ≥ 90, and 47.7% (83/174) were current or former smokers. Eighty-six patients (49.4%) achieved a good intracranial response, including 20 iCR and 66 iPR, whereas 88 patients (50.6%) achieved a poor intracranial response, including 42 iSD and 46 iPD. A total of 153 patients (87.9%) had adenocarcinoma; 38 (21.8%) had epidermal growth factor receptor (EGFR) mutations, and 52 (23%) developed liver metastases. Of the 174 patients, 52 (29.9%) received ICIs in combination with targeting therapy. Twenty-six patients (14.9%) received planned concurrent radiation therapy. Ninety-four patients (54.0%) had solitary BMs, and 80 patients (46.7%) had a maximum BM diameter < 1 cm. A comprehensive overview of the characteristics observed in both the training and validation groups is presented in **Table 1**. The clinical characteristics of the two cohorts were not significantly different (all *p* > 0.05).

**Table 1:** Characteristics of patients in the training and validation cohorts.

Characteristics	Training set (n = 122)	Validation set (n = 52)	P-value
Age, years*	60.00(52.00,65.00)	57.50(52.00,64.00)	0.607
Gender			0.669
Male	90 (73.8%)	36 (69.2%)	
Female	32 (26.2%)	16 (30.8%)	
KPS			0.227
≥90	45 (36.9%)	25 (48.1%)	
≤80	77 (63.1%)	27 (51.9%)	
Smoking history			0.920
Yes	59 (48.4%)	24 (46.2%)	
No	63 (51.6%)	28 (53.8%)	
Pathology			0.693
Squamous	16 (13.1%)	5 (9.62%)	
Adenocarcinoma	106 (86.9%)	47 (90.4%)	
EGFR			0.954
Negative or unknown	96 (78.7%)	40 (76.9%)	
Positive	26 (21.3%)	12 (23.1%)	
Clinical T stage			0.897
0,1,2	71 (58.2%)	29 (55.8%)	
3,4	51 (41.8%)	23 (44.2%)	
Clinical N stage			0.640
0,1,2	64 (52.5%)	30 (57.7%)	
3	58 (47.5%)	22 (42.3%)	
Liver involvement			1.000
No	94 (77.0%)	40 (76.9%)	
Yes	28 (23.0%)	12 (23.1%)	
Number of BMs			0.892
Solitary	65 (53.3%)	29 (55.8%)	
Multiple	57 (46.7%)	23 (44.2%)	
Max diameter of BMs			0.892
<1cm	57 (46.7%)	23 (44.2%)	
≥1cm	65 (53.3%)	29 (55.8%)	
LDH, U/L*	222.50(188.75,307.75)	218.00(176.50,263.75)	0.135
LYM,10^9/L*	1.39(1.04,1.73)	1.29(1.06,1.78)	0.986
NEUT,10^9/L*	4.31(2.94,5.53)	3.84(2.50,5.42)	0.273
NLR*	3.20(2.06,4.75)	3.01(1.97,4.08)	0.328
Combined targeting therapy			0.728
No	87 (71.3%)	35 (67.3%)	
Yes	35 (28.7%)	17 (32.7%)	
Concurrent brain radiotherapy			0.422
No	106 (86.9%)	42 (80.8%)	
Yes	16 (13.1%)	10 (19.2%)	
Intracranial response			0.691
Good (iCR+iPR)	62 (50.8%)	24 (46.2%)	
Poor (iSD+iPD)	60 (49.2%)	28 (53.8%)	

P-value of significant difference between training and validation cohorts. Abbreviations: KPS, Karnofsky Performance Status; EGFR, Epidermal Growth Factor Receptor; T, tumor; N, Node; BMs, brain metastasis; LDH, lactate dehydrogenase; NLR, Neutrophil to lymphocyte ratio; iCR, intracranial complete response; iPR, intracranial partial response; iSD, intracranial stable disease; iPD, intracranial progress disease. Data are numbers of patients, with percentages in parentheses. *Values refer to median (interquartile range).

Clinically significant features associated with poor intracranial response were liver metastases, EGFR mutations, and a KPS score ≤ 80. Conversely, a good intracranial response was associated with adenocarcinoma histopathology, concurrent brain radiotherapy, higher neutrophil counts, older age, and clinical stage N3 disease (**Supplementary Figure S1**). All the significantly selected clinical features were used for the clinical signature development.

### 3.2 Radiomic feature selection

A total of 1745 features were derived from each ROI. After conducting three steps to determine the optimal features for predicting the intracranial immunotherapy response, we ultimately identified 14 intratumoral and 17 peritumoral features that exhibited a significant potential association with the intracranial response. An overview of the LASSO feature selection process was provided in **Supplementary Figures S2A, B**, **S3A, B**. Based on the LASSO logistic regression model, the radiomic features that had a nonzero coefficient were shown in **Supplementary Figures S2C, D**, **S3C, D**, **Supplementary Table 1**.

### 3.3 Refinement and validation of signatures by constructing a nomogram

**Figure 2:**
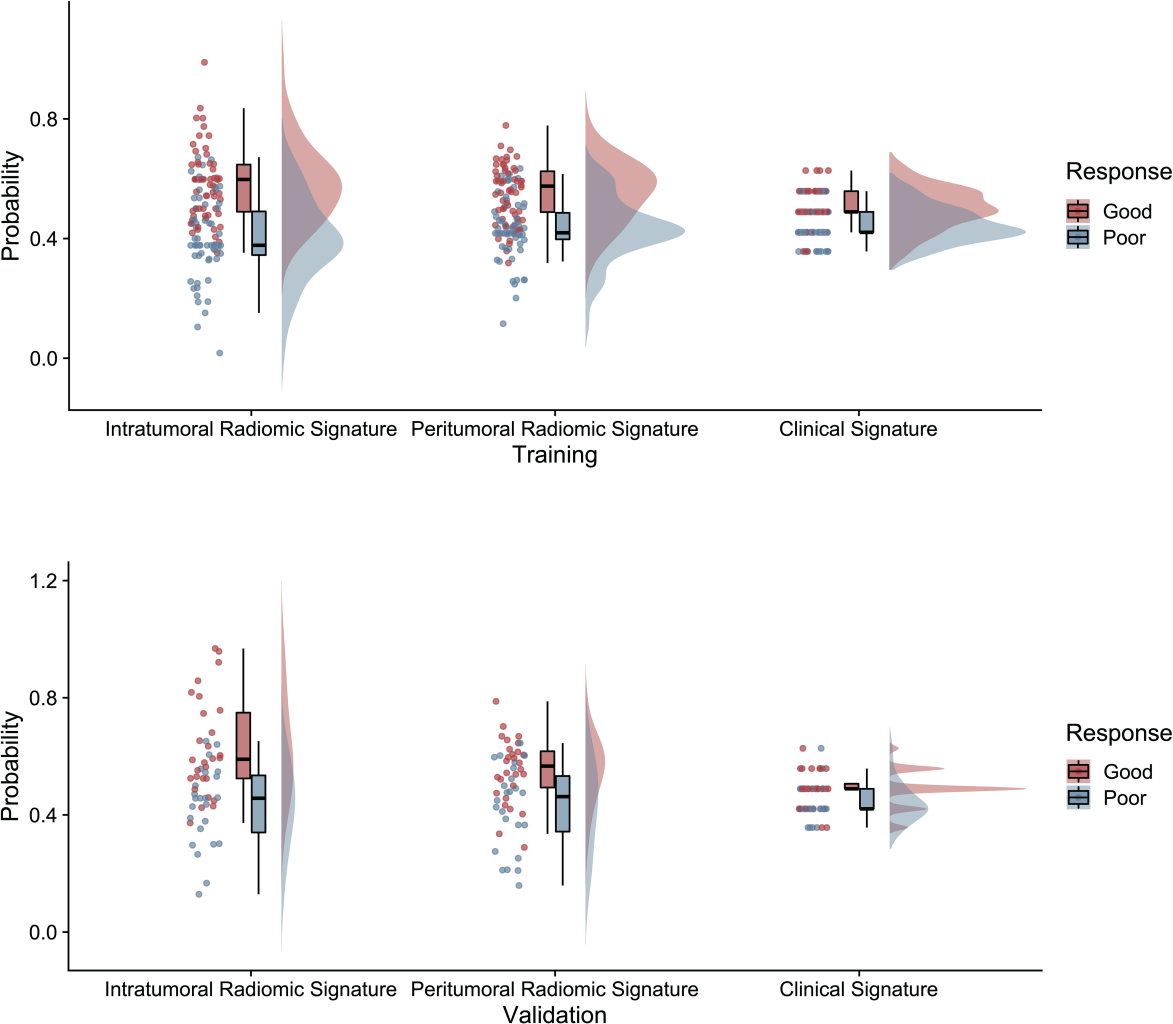
The raincloud plot visualizes the radiomics signature score, illustrating the spatial distribution and density of samples in both the training and validation sets of radiomics signatures.

**Figure 3:**
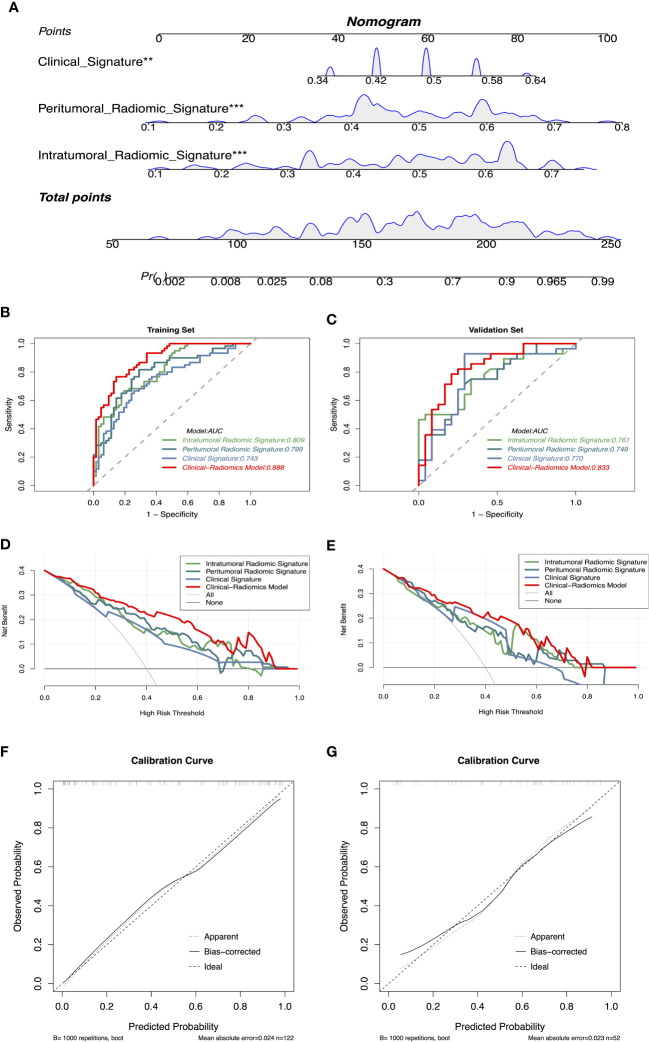
**(A)** CRN based on clinical and radiomics signatures, ** represents p < 0.01, *** represents p < 0.001. **(B, C)** Receiver operating characteristics curves demonstrate the accuracy in predicting the intracranial response of IRS, PRS, CS, and CRN in the training and validation sets. **(D, E)** Decision curve analysis demonstrates the clinical utility of different models for predicting the intracranial response in the training and validation sets. **(F, G)** Calibration curves of CRN for intracranial response prediction. CRN, Clinical-Radiomic nomogram; IRS, Intratumoral Radiomic Signature; PRS, Peritumoral Radiomic Signature; CS, Clinical Signature.

Based on the significant features associated with intracranial immunotherapy response in terms of intratumoral, peritumoral, and clinical factors, we used SVM to construct three predictive signatures: IRS, PRS, and CS. We observed that patients with distinct intracranial responses exhibited statistically significant differences in all three signatures within the training and validation sets, confirming their discriminative capacity. A raincloud plot was used to visually represent the varying sample distributions (**Figures 2A, B**). Moreover, multivariate linear regression analysis conducted on the training set revealed that IRS, PRS, and CS were independent predictors of the intracranial response (all *p* < 0.001). Consequently, we developed a predictive CRN model in the training cohort by integrating these three signatures (**Figure 3A**).

The CRN accurately predicted the intracranial response to immunotherapy, with AUC values of 0.888 (95% CI: 0.834–0.944) and 0.833 (95% CI: 0.720–0.946), in the training and validation sets, respectively. The IRS, PRS, and CS AUC values were 0.809 (95% CI: 0.735–0.884), 0.799 (95% CI: 0.720–0.879), and 0.743 (95% CI: 0.655–0.831), respectively, in the training set, and 0.761 (95% CI: 0.630–0.892), 0.749 (95% CI: 0.616–0.883), and 0.770 (95% CI: 0.630–0.911), respectively, in the validation set. The corresponding ROCs are shown in **Figures 3B, C**.

According to the DeLong test (**Supplementary Table 2**), CRN showed the best predictive with SPE, ACC, and PPV among the four models in the training set (SPE: 85.48%, ACC: 81.15%, PPV: 83.64%). In the validation set, CRN showed moderate predictive performance compared with the other models (SEN: 78.57%, SPE: 79.17%, ACC: 78.85%, PPV: 81.48%, NPV: 76.00%). Detailed statistical results of different models assessing the efficacy of intracranial immunotherapy are presented in **Table 2**. DCA showed that the CRN is a reliable and valuable tool for predicting intracranial response to immunotherapy (**Figures 3D, E**). Furthermore, both in the training and validation sets, a calibration curve was constructed and a Hosmer-Lemeshow test showed that the CRN aligned well with the actual observations (*p* > 0.05; **Figures 3F, G**).

**Table 2:** Performance of models for predicting intracranial response in patients treated with ICIs.

Training set	AUC (95%CI)	SEN (%)	SPE (%)	ACC (%)	PPV (%)	NPV (%)
IRS	0.809(0.735-0.884)	66.67	80.65	73.77	76.92	71.43
PRS	0.799(0.720-0.879)	81.67	70.97	76.23	73.13	80.00
CS	0.743(0.655-0.831)	66.67	74.19	70.49	71.43	69.70
CRN	0.888(0.834-0.944)	76.67	85.48	81.15	83.64	79.10
Validation set	AUC (95%CI)	SEN (%)	SPE (%)	ACC (%)	PPV (%)	NPV (%)
IRS	0.761(0.630-0.892)	46.43	100.00	71.15	100.00	61.54
PRS	0.749(0.616-0.883)	67.86	75.00	71.15	76.00	66.67
CS	0.770(0.630-0.911)	92.86	70.83	82.69	78.79	89.47
CRN	0.833(0.720-0.946)	78.57	79.17	78.85	81.48	76.00

AUC, area under the receiver operating curve; CI, confidence interval; SEN, sensitivity; SPE: specificity; ACC, accuracy; PPV, positive predictive value; NPV, negative predictive value; IRS, intratumoral radiomic signature; PRS, peritumoral radiomic signature; CS, clinical signature; CRN, clinical-radiomics nomogram.

### 3.4 Outcomes

The median follow-up was 12.6 months. The median iPFS (miPFS) was 7.9 months (interquartile range [IQR]:3.7–14.0 months), the median PFS (mPFS) was 7.0 months (IQR: 3.2–13.5 months), and the median OS (mOS) was 12.6 months (IQR: 7.2–20.4 months).

**Figure 4:**
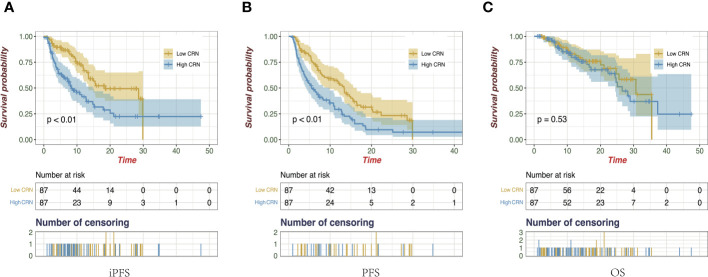
Kaplan-Meier survival curves of intracranial progression-free survival **(A)**, progression-free survival **(B)**, and overall survival **(C)** on the follow-up of all enrolled patients.

The Kaplan–Meier analysis revealed that patients who had a low score based on CRN (low CRN) compared with those who had a high score (high CRN) had a higher miPFS (18.43 vs 8.63 months; *p* < 0.001; **Figure 4A**) and mPFS (13.37 vs 5.50 months; *p* < 0.001; **Figure 4B**). However, there was no significant difference in the mOS observed between the low and high CRN groups (30.73 vs. 26.60 months; *p* = 0.53; **Figure 4C**).

## 4 Discussion

In this study, we developed and validated a radiomic model called CRN, which utilizes T1CE images to evaluate the intracranial reaction to immunotherapy in NSCLC patients with BMs. Additionally, our model accurately detected individuals who are at an elevated risk of experiencing intracranial progression. Pretreatment radiomics could potentially forecast the intracranial reaction of NSCLC patients with BMs to early immunotherapy, offering the possibility of personalized treatment guidance for high-risk individuals.

Recently, ICIs have made noteworthy clinical advances in patients with NSCLC by overcoming immunosuppressive signals in the tumor microenvironment. However, the response of BMs to ICIs remains unclear. Data from the KEYNOTE-024, CheckMate 017, and 057 trials did not demonstrate any significant disparity in survival rates between patients with BMs who received ICI treatment and those who underwent chemotherapy (10, 20, 21). In contrast, the KEYNOTE-189 and OAK trials showed that patients with BMs treated with ICIs experienced extended OS than those who underwent chemotherapy (9, 22). In the past, the brain has been regarded as a privileged organ within the immune system because of the presence of the blood-brain barrier (BBB), which shields it from infiltration by immune cells (23). However, preclinical models have demonstrated that ICIs exert their effects by activating T cells capable of crossing the BBB (24). The potential mechanism underlying the intracranial response to ICIs may be attributed to the distinct microenvironment in BMs compared with that of primary tumors (25–27), and the prevailing immunosuppressive immune microenvironment in BMs (7, 28).

There is a requirement for the development of non-invasive biomarkers to accurately predict the intracranial response and prognosis to ICIs in patients with NSCLC. The level of PD-L1 expression is the only biomarker that has received approval for immunotherapy; however, its utility remains unclear (13, 29–33). TILs and TMB have also been explored as potential biomarkers for immunotherapy, but do not have a significant association with the intracranial response or prognosis (11–13). The inflammatory microenvironment of BMs varies considerably, with the presence of TILs ranging from complete absence to dense infiltration (34). Furthermore, the composition of TIL subtypes within this microenvironment varies, encompassing stimulated cytotoxic T cells, immune-suppressing T cells, and fatigued T cells (35). A previous study investigated TMB and T-cell richness in BMs (27). The results showed that the TMB was significantly increased in the BM samples compared to the primary NSCLC specimens. In contrast, the anticipated tumor neoantigen burden did not show any notable variation, while the richness of T-cell clones was significantly reduced in the BM samples compared to the primary samples. In summary, the search for new and robust biomarkers for predicting the intracranial response of BMs to ICIs remains challenging because of the inherent difficulty in obtaining BM biopsies and the significant heterogeneity and diversity of the immune landscape within the tumor microenvironment between BMs and the primary lesion.

Recently, several research studies have investigated the possible practicality of radiomic features extracted from images of malignant tumors and their correlation with immunotherapy response or outcome (15, 16, 36–39). Bhatia et al. (15) extracted radiomic features from MRI images and investigated the correlation between these features and OS in patients with melanoma BMs treated with ICIs. The findings indicate that radiomic features derived from MRI images are significantly correlated with patient OS, suggesting that radiomic features may serve as effective biomarkers for assessing treatment response and predicting patient prognosis. Li et al. (16) found that prognostic radiomic features from MRI were closely correlated with tumor-infiltrating macrophages and patient clinical outcomes in gliomas, which revealed a relationship between radiomic biomarkers and the tumor microenvironment. Sun et al. (40)discovered that imaging biomarkers may serve as a valuable tool for estimating the count of CD8 cells and for prognosticating the clinical responses of patients undergoing immunotherapy. However, a radiomic prediction model to evaluate the intracranial response to ICIs in patients with NSCLC BMs is still under development. In this study, the developed radiomic model could accurately predict the intracranial response and enhance clinical decision-making for ICI treatment in patients with NSCLC with BMs. Despite the observed correlation between radiomic biomarkers and patient survival, it appears that there are currently no systematic studies that thoroughly investigate the direct relationships between specific radiomic features and their biological implications further investigations are warranted to delve into the intrinsic relationship between radiomics and the BM microenvironment (41).

This study has some limitations. First, it was a retrospective study, and despite including data from two centers, the overall size of the sample was comparatively limited. To prevent fitting risks due to the small data volume, all features we selected using LASSO with 5-fold cross-validation via minimum criteria, which can reduce unnecessary model complexity and assist in variable selection when the number of samples is small size, thereby mitigating the risk of overfitting. Incorporating a subset of patient data from external institutions as an independent validation set also mitigated the risk of overfitting. Second, the analysis was limited to baseline radiomic features before treatment owing to the absence of enhanced MRI scans at a certain time point during treatment. Thirdly, the nomogram could not predict OS, because some patients had received other therapy post-immunotherapy progression which may influence the result of predicted OS. These limitations should be fully taken into account in future prospective research to build predictive models with generalizability and reliability. To validate these models, it is necessary to conduct extensive prospective research across multicenters.

## 5 Conclusion

In conclusion, we constructed a radiomic nomogram using MRI as a biomarker to predict the intracranial response to ICIs in patients with NSCLC with BMs. The nomogram was also predictive of iPFS and PFS. The nomogram offers personalized treatment guidance for high-risk individuals before they receive immunotherapy.

## Data availability statement

The original contributions presented in the study are included in the article/**Supplementary Material**. Further inquiries can be directed to the corresponding author.

## Ethics statement

The studies involving humans were approved by Shandong Cancer Hospital and Institution and Cheeloo Hospital Research Ethics Committee. The studies were conducted in accordance with the local legislation and institutional requirements. Written informed consent for participation was not required from the participants or the participants’ legal guardians/next of kin in accordance with the national legislation and institutional requirements.

## Author contributions

JX: Conceptualization, Data curation, Formal Analysis, Investigation, Methodology, Project administration, Resources, Software, Validation, Visualization, Writing – original draft, Writing – review & editing. PW: Conceptualization, Data curation, Methodology, Validation, Writing – review & editing. YL: Data curation, Writing – review & editing. XS: Data curation, Writing – review & editing. TY: Data curation, Writing – review & editing. JY: Conceptualization, Funding acquisition, Methodology, Project administration, Resources, Supervision, Writing – review & editing. FT: Conceptualization, Data curation, Formal Analysis, Funding acquisition, Investigation, Methodology, Project administration, Resources, Software, Supervision, Validation, Visualization, Writing – review & editing.
