# Supplementary material for: Development and validation of an MRI-Based nomogram to predict the effectiveness of immunotherapy for brain metastasis in patients with non-small cell lung cancer
Source: Front Immunol. 2024 Apr 15;15:1373330. doi: 10.3389/fimmu.2024.1373330 (PMC11057328; doi:10.3389/fimmu.2024.1373330)
Supplement: Supplementary file 1 [file DataSheet_1.docx]

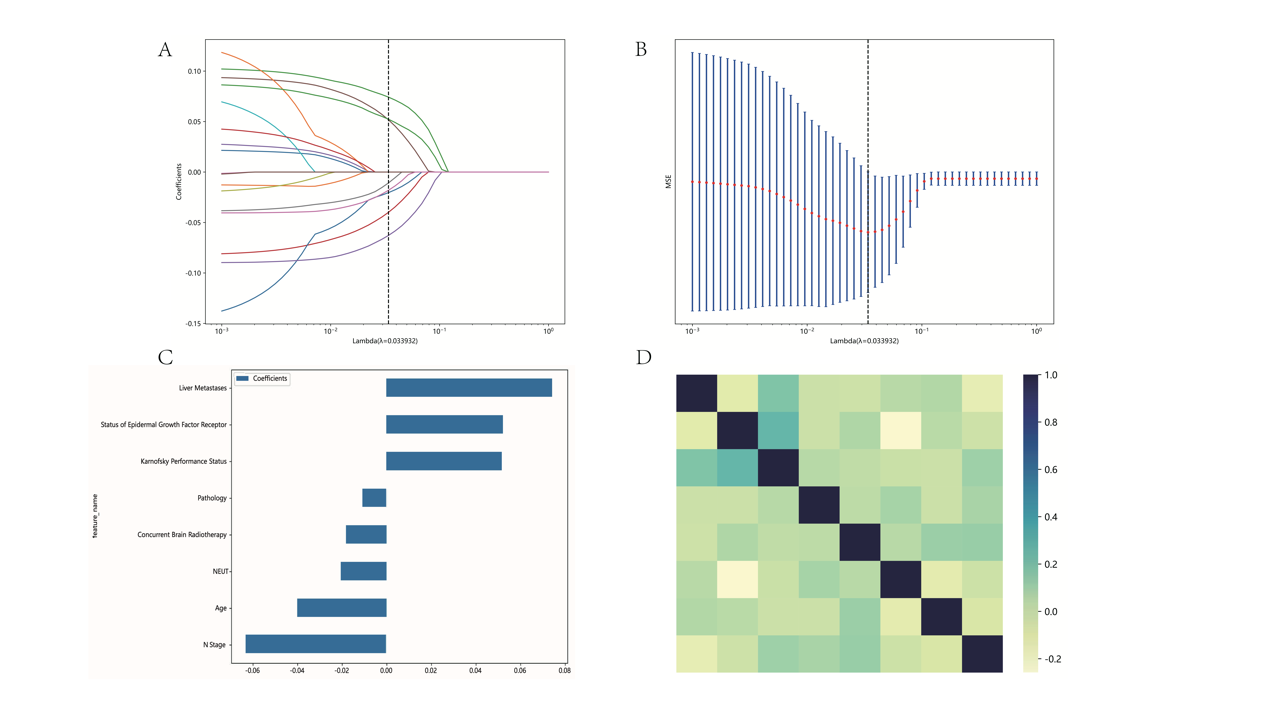


**Supplementary Figure 1** (A) The LASSO coefficient profiles of the 8 clinical features. (B) Selection of tuning parameter (λ) by the LASSO model using 5-fold cross-validation via minimum criteria. The x-axis shows lambda, and the y-axis shows the mean squared error (MSE). (C) Histogram showing the role of selected optimal features with LASSO coefficients that contributed to the developed clinical signature. (D) The heatmap shows the correlation coefficient between the clinical features selected by the LASSO.


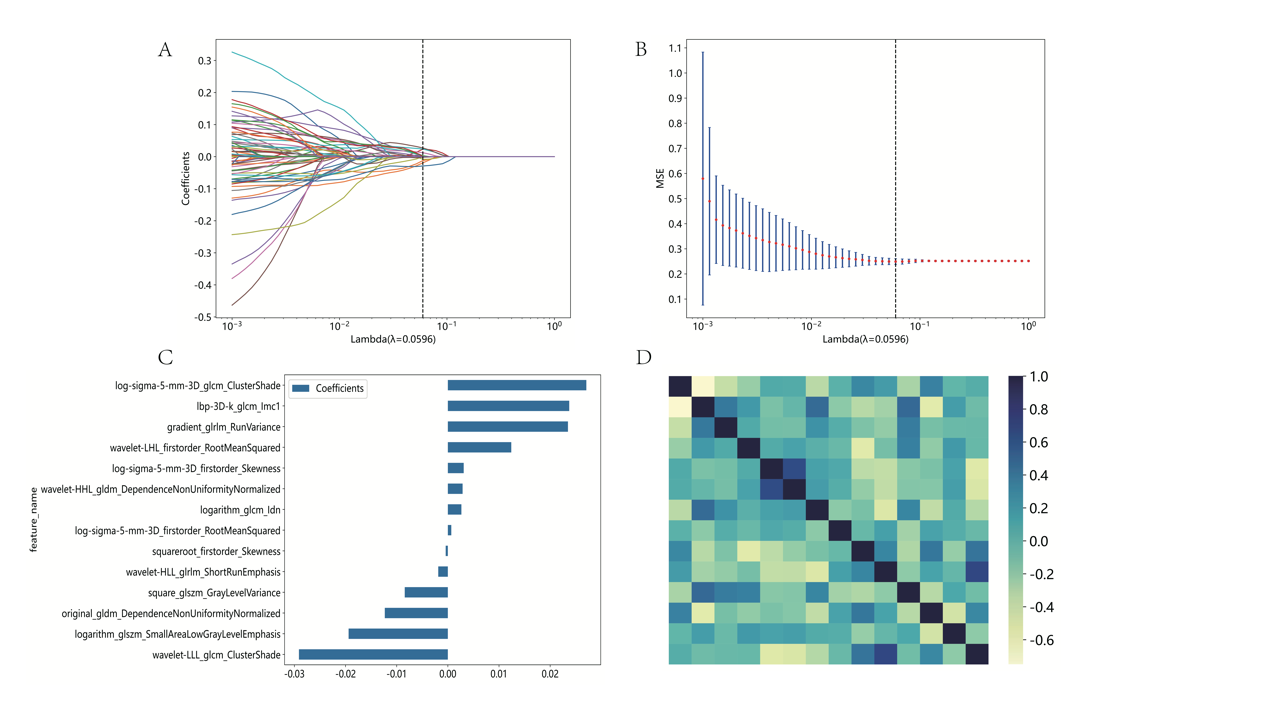


**Supplementary Figure 2** (A) The LASSO coefficient profiles of the 14 intratumoral radiomic features. (B) Selection of tuning parameter (λ) by the LASSO model using 5-fold cross-validation via minimum criteria. The x-axis shows lambda, and the y-axis shows the mean squared error (MSE). (C) Histogram showing the role of selected optimal features with LASSO coefficients that contributed to the developed intratumoral radiomic signature. (D) The heatmap shows the correlation coefficient between the intratumoral radiomic features selected by the LASSO.


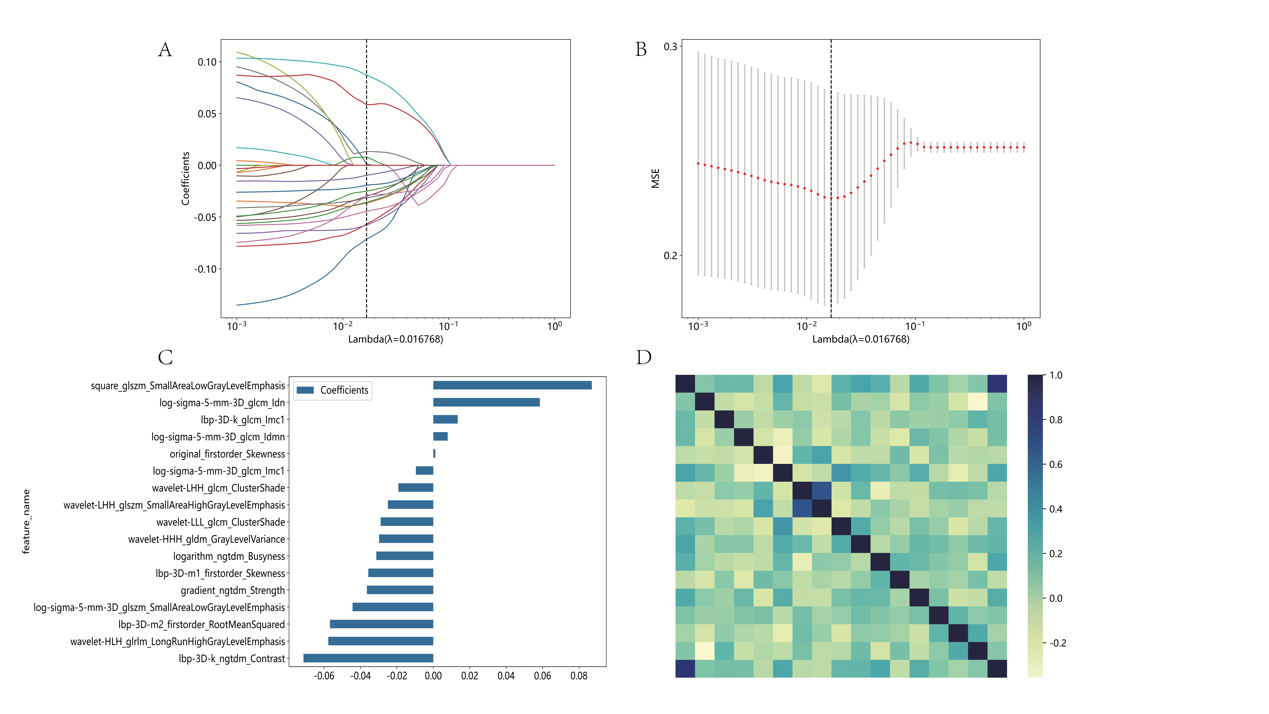


**Supplementary Figure 3** (A) The LASSO coefficient profiles of the 17 peritumoral radiomic features. (B) Selection of tuning parameter (λ) by the LASSO model using 5-fold cross-validation via minimum criteria. The x-axis shows lambda, and the y-axis shows the mean squared error (MSE). (C) Histogram showing the role of selected optimal features with LASSO coefficients that contributed to the developed peritumoral radiomic signature. (D) The heatmap shows the correlation coefficient between the peritumoral radiomic features selected by the LASSO.

| **Supplementary Table 1** Radiomic features selected by LASSO | |
| --- | --- |
| Intratumoural radiomic features | original_gldm_DependenceNonUniformityNormalized |
|  | gradient_glrlm_RunVariance |
|  | lbp-3D-k_glcm_Imc1 |
|  | log-sigma-5-mm-3D_firstorder_RootMeanSquared |
|  | log-sigma-5-mm-3D_firstorder_Skewness |
|  | log-sigma-5-mm-3D_glcm_ClusterShade |
|  | logarithm_glcm_Idn |
|  | logarithm_glszm_SmallAreaLowGrayLevelEmphasis |
|  | square_glszm_GrayLevelVariance |
|  | squareroot_firstorder_Skewness |
|  | wavelet-LHL_firstorder_RootMeanSquared |
|  | wavelet-HLL_glrlm_ShortRunEmphasis |
|  | wavelet-HHL_gldm_DependenceNonUniformityNormalized |
|  | wavelet-LLL_glcm_ClusterShade |
| Peritumoural radiomic features | original_firstorder_Skewness |
|  | gradient_ngtdm_Strength |
|  | lbp-3D-m1_firstorder_Skewness |
|  | lbp-3D-m2_firstorder_RootMeanSquared |
|  | lbp-3D-k_glcm_Imc1 |
|  | lbp-3D-k_ngtdm_Contrast |
|  | log-sigma-5-mm-3D_glcm_Idmn |
|  | log-sigma-5-mm-3D_glcm_Idn |
|  | log-sigma-5-mm-3D_glcm_Imc1 |
|  | log-sigma-5-mm-3D_glszm_SmallAreaLowGrayLevelEmphasis |
|  | logarithm_ngtdm_Busyness |
|  | square_glszm_SmallAreaLowGrayLevelEmphasis |
|  | wavelet-LHH_glcm_ClusterShade |
|  | wavelet-LHH_glszm_SmallAreaHighGrayLevelEmphasis |
|  | wavelet-HLH_glrlm_LongRunHighGrayLevelEmphasis |
|  | wavelet-HHH_gldm_GrayLevelVariance |
|  | wavelet-LLL_glcm_ClusterShade |
|  |  |

| **Supplementary Table 2** Delong test between different models. | | |
| --- | --- | --- |
| Models | | p-value |
| CRN | IRS | 0.005 |
| CRN | PRS | 0.008 |
| CRN | CS | 0.0005 |
| Note: IRS: intratumoral radiomic signature; PRS: peritumoral radiomic signature; CS: clinical signature; CRN: clinical-radiomics nomogram. | | |
